# Our Women, Warriors, and Workers

**Published:** 1916-12-23

**Authors:** 


### THE MATRONS' AND SISTERS' DEPARTMENT

### OUR WOMEN, WARRIORS, AND WORKERS.

The war, with all its horrors, has still been a great awakener and builder. From the very outset it has aroused British women everywhere to an activity and continuous effort for which history contains no equivalent. British nurses have everywhere done credit to their training and their country, whilst the large army of untrained women who have given their services in the humblest capacities on war service have won for themselves a name which will live in history. The Principal Matrons throughout the country have taken upon themselves onerous positions entailing double, and sometimes three times the amount of responsibility and labour which they have ever been called upon to carry. British matrons have indeed shown a prowess for work and a conscientious awakening to duty which is as notable as it is admirable. Indeed, their example has had a stimulating effect throughout the whole field occupied by women workers, an increasing number of whom are coming to recognise, that, they are endowed with a capacity for work, and good work too, of which they had no idea before the war.

Good work, done with a total forgetfulness of self, produces splendid results, not the least of which is a recognition on the part of the worker that, such work takes care of her and frees her from worries, which aforetime, may have made her anxious. As an interested witness of all these things we have often felt that, those of our warriors who have abstained from marriage until the war is over should have the finest opportunity to secure a helpmeet of the best, and find her amongst the army of women who have proved themselves to be the possessors of remarkable capacity and splendid character. When the victory is won and peace comes, accompanied by adequate guarantees that the world will in future be protected from similar crimes and iniquities to those perpetrated by the reckless, renegade rulers of certain nations, with freedom for the unfortunate slaves they have enchained under their military system for slaughter and destruction, it is hoped that God-fearing, decent people will enter upon their own, and that the world will be rid for ever of the worst and most degenerate type of humanity it has ever produced.

With the dawn of the New Era and the re-establishment of Christ's Kingdom upon Earth, life on this planet should assume a brightness and vitality which may effect changes for the better in the circumstances of every class and of both sexes. These changes must bring with them the fulfilment of ideals hitherto felt to be impossible of attainment. All honour to the women who have forgotten self and devoted all their energies to the relief and recovery of the wounded warriors, the comfort and solace of the mourners and the bereft, and the awakening of the recognition of the spiritual nature in man and woman, which had almost lost its reality with most of the people. Such women will attain a position which should give their sex equality with their "lords and masters," and the power to take their part in making the world sweeter and better than it was when they entered it. And what of the "lords and masters," as they were called by a former generation? If we may judge by the wonderful changes which have come over the men, who have fought and are fighting the battle of Christ in this war, they will return to their homes worthy mates, companions, defenders, and friends of the noble women, who have done their part at home with such splendid spirit and ennobling results.

It is our privilege to-day to wish in heartfelt sincerity a Blessed Christmas and a Bright New Year to all our readers, who, in a sense which is probably not true to an equal extent in regard to the readers of any other British newspaper, have been for the most part members of the noble army of men and women to whom we have just referred. Surely this last year has been blessed to them in a sense never probably attained before. It has been full of opportunities to render personal service continuously to the sailor and soldier in their hour of direst trial, suffering, and anxiety. Who can appraise the value of the sympathy, kindliness, skill, and devoted attention, which every type of hospital workers, from the highest to the lowest, has untiringly and gladly rendered to the patients, both military and civil? And then, too, the awakening in the sailor and the soldier has often been transferred to his attendants. It has raised the hopes of many anxious minds. It has brought them consolation through a recognition of their Heavenly Father, and the uplifting and purification which have come to many people in this last twelve months, with a closer apprehension of Christ's teaching and all it means and has ever meant for humanity.

One special word we would offer to all trained nurses throughout the hospital world. To you 1916 has produced the opportunity to form yourselves into one great profession, to establish upon a lasting and adequate basis a Great College of British Nursing, to raise and fix a high standard of education, of independence, noble aspirations and authority for every British Nurse, who is proud of her profession and determined to sustain its reputation everywhere throughout the world. We have had a splendid answer to our efforts to rally British Nurses everywhere, to a sense of the responsibility which attaches to every one of them in these great days of opportunity. Every qualified nurse owes it to herself immediately to Register with the College of Nursing, and, having done so, to make it her business, in the coming year, to work continuously to bring all her fellow-nurses to adopt this course. If they do British Nurses will have a security and a position which will make them famous, happy and strong, to an extent not yet equalled, by probably any type of women workers, anywhere in the world.

